# Clinical and Patient-Reported Outcomes of Laceback Ligature in Fixed Orthodontic Treatment: Protocol for a Randomized Controlled Trial

**DOI:** 10.2196/88793

**Published:** 2026-07-16

**Authors:** Loon Han Tan, Siti Hajjar Nasir, Joo Ming Cheong, Kumeran Mohan

**Affiliations:** 1 Department of Orthodontics, Kulliyyah of Dentistry, International Islamic University Malaysia, Jalan Sultan Ahmad Shah, Bandar Indera Mahkota, Kuantan, Pahang, 25200, Malaysia, 60197297546

**Keywords:** orthodontic wires, laceback, treatment outcome, orthodontic tooth movement, split-mouth study, patient reported outcome measures

## Abstract

**Background:**

Laceback ligatures are passive stainless-steel auxiliaries tied from the first permanent molar hook to the canine bracket during the alignment phase of orthodontic fixed appliance treatment, predominantly in premolar extraction cases. Despite equivocal evidence on their effectiveness in controlling anterior tooth position, lacebacks are routinely used to stabilize flexible nickel-titanium archwires against masticatory forces. Their effects on 3 clinically important outcomes, oral hygiene, appliance complication rates, and patient-reported pain, have never been investigated in a randomized trial, representing gaps explicitly identified in a prior systematic review.

**Objective:**

This study aims to evaluate the clinical and patient-reported outcomes of passive laceback ligatures during the alignment phase of orthodontic fixed appliance treatment, specifically oral hygiene status, archwire and laceback complication rates, and pain.

**Methods:**

This is a single-center, single-blind, split-mouth randomized controlled trial conducted at the Orthodontic Clinic, International Islamic University Malaysia. Eligible participants are patients aged 15 years and older, medically fit with Basic Periodontal Examination scores of 0‐2, requiring full fixed appliance treatment with bilateral premolar extractions and no prior orthodontic treatment. Those with craniofacial deformities, systemic illness, or disability affecting oral hygiene compliance are excluded. Each participant’s oral cavity is randomly allocated using a minimization method, so that one side receives a passive 0.010-inch stainless-steel laceback from the first molar hook to the canine bracket, while the contralateral side receives identical standard fixed appliance treatment (McLaughlin, Bennett, and Trevisi prescription brackets, 0.014-inch superelastic nickel-titanium archwires, and elastomeric modules) without laceback. Outcomes are assessed at baseline (T0), 4 weeks (T1), and 8 weeks (T2) after laceback placement. The primary outcome is the orthodontic plaque index. Secondary outcomes include archwire and laceback complication frequencies and pain assessed by visual analog scale area under the curve. A total of 38 participants provided 85% power to detect a 1-unit orthodontic plaque index difference (*α*=.05). Mixed-model repeated-measures analysis is applied to plaque scores; paired parametric or nonparametric tests to other outcomes. Plaque and archwire assessors are blinded to treatment allocation.

**Results:**

Ethical approval and funding were secured in January 2026. Participant recruitment commenced in February 2026 with a target completion of June 2027. Pretrial examiner calibration achieved satisfactory agreement (interrater Cohen kappa=0.801-0.804; intrarater reliability=0.806-0.862). As of paper submission, 24 participants have been enrolled, and 14 have completed all data collection visits, with no protocol deviations recorded. Primary analysis is scheduled for October 2027; the results are targeted for submission by June 2028.

**Conclusions:**

This protocol describes the first randomized controlled trial to simultaneously evaluate oral hygiene, appliance complication rates, and patient-reported pain associated with laceback use during orthodontic alignment. The split-mouth design controls for interparticipant confounders, and the incorporation of patient-reported outcomes will ensure that findings directly support evidence-based, patient-centered orthodontic practice.

## Introduction

Orthodontic fixed appliances are widely used in orthodontic treatment to correct malpositioned teeth. It involves bonding brackets to the surfaces of the teeth and ligating each bracket to archwires. The preadjusted edgewise appliance, commonly referred to as the straight-wire appliance, incorporates bracket-specific prescriptions for tooth movements in 3 dimensions, thereby reducing the need for complex archwire bending [1]. During the initial alignment and leveling phase, the inherent mesial crown tip incorporated into anterior bracket prescriptions of the preadjusted edgewise appliance may result in forward displacement of the canine crown and consequent proclination of the anterior labial segment. This tendency is particularly problematic in premolar extraction cases, where unsupported spans of archwire and the absence of tooth contact distal to the canines create conditions favoring forward drift of the anterior dentition. To counteract this undesirable tooth movement, laceback ligatures were originally proposed by McLaughlin and Bennett as part of the McLaughlin, Bennett, and Trevisi (MBT)–preadjusted edgewise system. Lacebacks are stainless-steel ligatures, typically 0.009 or 0.010 inches in diameter, tied in a figure-of-eight configuration beneath the main archwire from the hook of the first permanent molar bracket to the canine bracket of the same quadrant [2].

Despite the conceptual rationale for laceback use, the clinical evidence supporting its effectiveness in controlling anterior tooth position remains limited and equivocal. Some researchers reported retroclination of incisors when lacebacks were used [3]. However, usage of laceback is also accompanied by loss of anchorage via mesial migration of molars, indicating a compromise of posterior anchorage rather than a benefit [4]. A systematic review and meta-analysis concluded that the anteroposterior position of incisors and molars was not significantly affected by the use of laceback during the initial phase of orthodontic fixed appliance therapy [5].

The practice of using laceback has not received widespread approval. Nowadays, some clinicians still use lacebacks to support the flexible archwires during early alignment phases of treatment, especially from masticatory forces [6]. Nickel-titanium archwires are superelastic and flexible wires that are routinely used during the alignment stages. However, these wires are characteristically soft and prone to dislodgement from terminal molar tubes or fracture across long, unsupported spans at premolar extraction sites [7]. In order to mitigate such complications, mechanical devices such as bumper sleeves, cinch backs, crimpable stops, and composite stops may be placed at the distal ends of archwires to prevent dislodgement [8]. However, no trials have been conducted to verify this archwire-protective role of lacebacks.

Beyond their purported role in archwire stabilization, lacebacks themselves could be prone to complications such as breakage, dislodgement, and mucosal ulceration [9,10]. Laceback-related complications represent unplanned orthodontic emergencies that necessitate additional clinical appointments and, in general, may be associated with treatment delays. While the overall incidence of appliance breakage during fixed orthodontic therapy has been characterized in recent studies [11,12], evidence specifically addressing the frequency and clinical implications of laceback-related complications remains very limited.

The effect of laceback usage on patient oral hygiene represents a particularly significant and previously unexplored concern. Orthodontic fixed appliances are established plaque-retentive environments that impede effective plaque removal. The figure-of-eight configuration of lacebacks, positioned beneath the main archwire across the buccal segment, creates additional plaque-retentive surfaces and physical obstructions that may further impair toothbrushing efficacy. This can potentially exacerbate enamel demineralization, gingivitis, and the long-term risk of caries and periodontal disease [13]. This concern is amplified by the fact that lacebacks are predominantly applied during the early phase of orthodontic therapy, precisely when patients are still adapting to oral hygiene maintenance around fixed appliances and plaque accumulation is most rapid [14]. Despite this clinically plausible risk, the specific impact of lacebacks on plaque levels and periodontal health has not been studied, as explicitly noted in the systematic review by Fleming et al (2013) [5].

Pain and discomfort represent another important dimension of concern regarding laceback use. Orthodontic pain is the most frequently reported adverse effect of fixed appliance therapy, experienced by up to 90% of patients and particularly pronounced during the initial stages of treatment when orthodontic forces are first applied [15]. Soft tissue trauma from orthodontic appliances, including mucosal ulceration caused by bracket edges, archwire ends, and other auxiliaries, is a common and often painful complication that significantly impacts patient comfort and treatment compliance beyond the discomfort arising from periodontal ligament mechanoreceptors [16]. Pain is now recognized as a critical patient-reported outcome (PRO) that influences treatment compliance, appointment adherence, and overall patient satisfaction [17]. The inclusion of a laceback introduces an additional appliance component that may contribute to soft tissue irritation and pain during the initial adaptation period and may theoretically amplify force transmission to the canine and molar. However, no clinical study has yet examined whether lacebacks are associated with greater pain levels during the alignment phase of fixed appliance treatment.

Currently, despite equivocal evidence regarding the effectiveness of laceback in controlling forward tipping of anterior teeth and risk of posterior anchorage loss, laceback is still routinely used by clinicians in selected cases. In view of the paucity of data on oral hygiene status and patient-reported outcomes following laceback usage, this research aims to evaluate the clinical and PROs of laceback usage during the alignment phase of orthodontic fixed appliance treatment. The specific objectives are to assess the effect of laceback on a patient’s oral hygiene, to investigate the effect of laceback on the frequency of archwire complications, to determine the frequency of laceback complications, and to study the effect of laceback on pain. It is hoped that the results obtained may identify treatments that are both clinically effective and acceptable to patients, as well as provide supportive evidence for clinicians to judiciously weigh the risks and benefits of using lacebacks. The null hypothesis is that there will be no statistically significant difference in clinical and PROs between laceback (intervention) and no laceback (control) groups during the alignment phase of orthodontic fixed appliance treatment.

## Methods

### Study Design

This is a single-center, single-blind, split-mouth randomized controlled trial with a 1:1 allocation ratio for the 2 groups (left and right sides of the oral cavity). In this study, either the left or right side of each participant’s oral cavity is randomly assigned to receive a laceback, while the contralateral side serves as the control (no laceback). The study is aimed to fulfill as many items as possible in the CONSORT (Consolidated Standards of Reporting Trials) 2025 checklist for reporting randomized controlled trials, and the SPIRIT (Standard Protocol Items: Recommendations for Interventional Trials) 2025 checklist for preparing protocols of randomized controlled trials [18,19].

### Clinical Trial Registration

This trial has been registered at ClinicalTrials.gov (NCT07039071), where the protocol and the intended statistical analysis plan can be accessed.

### Study Participants

The target population is orthodontic patients from the Postgraduate Orthodontic Clinic of the Kulliyyah of Dentistry, International Islamic University Malaysia (IIUM). To ensure adequate enrollment, the principal investigator will be actively screening potential participants from the list of orthodontic patients receiving treatment at IIUM. The eligibility criteria are mentioned in Textbox 1.

Textbox 1. **Eligibility criteria.**

***Inclusion criteria***

**Patient factors**

- Age 15 years and older.
- Medically fit and well.
- Good oral hygiene—no dental caries; Basic Periodontal Examination score 0, 1, or 2 in all sextants.

**Orthodontic factors**

- Full upper and lower arch orthodontic fixed appliance.
- Extraction of bilateral upper and lower permanent first or second premolars at least 2 weeks before bond-up.
- No previous orthodontic treatment.

***Exclusion criteria***

- Craniofacial deformities.
- Mental or physical disability leading to impaired manual dexterity and difficulty comprehending and following oral hygiene instructions.
- Underlying chronic medical illness, oral diseases.

***Withdrawal criteria***

Participants are free to withdraw anytime during the period of study. Reasons for withdrawal will be noted down. Possible criteria of withdrawal include:

- Serious adverse events arising from laceback, which is very unlikely as it has not been reported in literature.
- Participant not following study procedures.
- Participant unwilling to continue orthodontic treatment.

An intention-to-treat analysis is used, whereby data from all participants is analyzed irrespective of any subsequent withdrawal or protocol deviation to ensure that the results will reflect the effects of laceback in a real-world setting, while maintaining the study power because no participants are omitted from the analysis [20,21]. Repeated measures data for plaque score and pain score will be analyzed using a mixed model for repeated measures (MMRM), which accounts for within-participant correlation and handles missing data without formal imputation under the missing at random assumption [22].

### Ethical Considerations

This study adheres to the ethical principles set out in the Declaration of Helsinki and the Malaysian Good Clinical Practice Guidelines. Ethical approval has been granted by the IIUM Research Ethics Committee (IREC-2026‐582) prior to the commencement of any recruitment activities. Any protocol deviation will be reported to the IIUM Research Ethics Committee. The study’s procedures, risks, and benefits are clearly explained by the principal investigator to both the patient and parent or guardian, and they are encouraged to ask questions. They also received a patient information sheet to take home and review before deciding whether to participate. Those who agreed to participate are required to sign the appropriate forms: participants younger than 18 years complete an assent form, and their parent or legal guardian signs the informed consent form, whereas participants older than 18 years sign the informed consent form. Participation in the study is entirely voluntary. No payments will be given for participation, and there are no costs involved in joining the study. All personal data are kept confidential in a password-protected laptop, and no participant’s identifiable data are mentioned in publications or presentations. Permission will be obtained from the Director General of Health before any planned publication and presentation of trial results.

### Sample Size Calculation

The sample size is determined using G*Power (Version 3.1.9.6, Heinrich Heine University, Düsseldorf, Germany). To assess the effect of laceback on a patient’s oral hygiene, a previous split-mouth study using the Orthodontic Plaque Index (OPI) to record plaque score on orthodontic patients will be used in this study [23]. By referring to the SEM and sample size (n) reported in that study, the SD of OPI is calculated to be 1.88 by applying the formula $SD=SEM\times \sqrt{n}$. A difference of 1 point in OPI was deemed clinically meaningful to be detected. A total of 34 participants are needed for 68 sites (34 laceback and 34 no laceback), which would give a power of 85% with a significance level of 0.05 to detect a 1-unit difference in OPI score. Assuming a 10% dropout rate, 38 participants are recruited, providing a total of 76 sites (38 laceback and 38 no laceback).

### Study Conduct

#### Randomization: Sequence Generation, Allocation Concealment, Implementation

Placement of laceback ligatures is randomly allocated to the participants’ left or right side. This study intends to balance 5 prespecified covariates between the laceback and no laceback groups:

1. Upper first premolar extraction
2. Upper second premolar extraction
3. Lower first premolar extraction
4. Lower second premolar extraction
5. Side of allocation (number of participants receiving laceback on the left vs right side)

The method of randomization used is the minimization method (also known as “covariate adaptive randomization”) [24]. This is because minimization achieves a better balance than stratified randomization when sample sizes are small (<100 participants) and several prognostic factors (≥3) need to be controlled simultaneously [25]. Therefore, this study uses a 2-stage randomization strategy: simple randomization, followed by minimization.

#### Simple Randomization

For the first 10 participants, simple randomization is applied to ensure an equal number of participants receive laceback intervention allocated to the left side (n=5) and to the right side (n=5) [26]. The random sequence is generated by an independent coinvestigator not directly involved in participant recruitment, using computer-generated random numbers in Microsoft Excel (2023). Allocation concealment is maintained using sequentially numbered, opaque, sealed envelopes containing the allocation card written “left” or “right.” These are kept secure in a separate box to prevent foreknowledge by the treating clinician and principal investigator. Whenever a new participant is recruited, an independent dental nurse is tasked to open the subsequent envelope to reveal the side allocated for laceback ligature. The cumulative counts of each covariate on the laceback and no laceback group for the first 10 participants are recorded.

#### Minimization

For the remaining 28 participants, allocation is determined using the minimization method. First, each participant’s finalized extraction pattern (left and right, upper and lower premolar type) is recorded. Overall, 2 hypothetical allocations are then evaluated: laceback on the left or laceback on the right. For each option, an imbalance score is calculated as the sum of absolute differences between the 2 trial arms across the 5 covariates, based on the cumulative allocations of all previously enrolled participants. The option with the lower imbalance score is considered the favored allocation [24,27].

**Figure 1.**
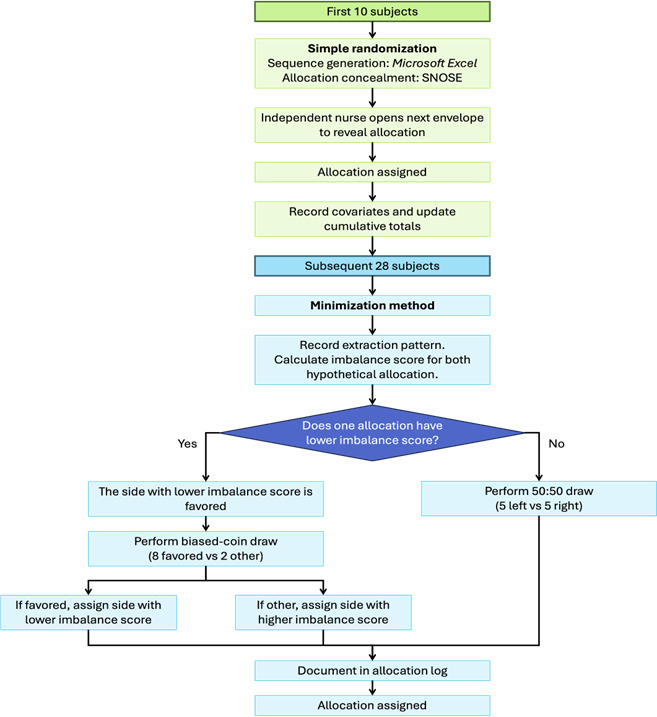
Randomization protocol. SNOSE: Sequentially numbered, opaque, sealed envelopes.

The next step involves performing a biased-coin draw, whereby the favored side is allocated with a probability of *P*=.8, and the other side with a probability of *P*=.20 [28,29]. The purpose of introducing this random element is to reduce the predictability of the next assignment while intentionally biasing the allocation toward the arm with a lower imbalance score [27,30]. To achieve this, a fully opaque container holds 10 identical slips: 8 “favored” slips and 2 “other” slips. If the research assistant draws a “favored” slip, the minimization-favored allocation is selected (ie, the option with lower imbalance score). If an “other” slip is drawn, the opposite allocation is applied (ie, the option with the higher imbalance score). In the event that the imbalance scores for left and right are identical, a 50:50 draw (5 “left” and 5 “right” slips) will be performed instead. All allocations are documented in an allocation log, including the participant’s extraction pattern, preallocation cumulative covariate totals, imbalance scores for both options, slips drawn, and final assignment. Allocation concealment is preserved until the moment of assignment. The randomization protocol is illustrated in Figure 1.

### Intervention

On one side that is randomly allocated, passive laceback is placed on the maxillary and mandibular arches prior to ligating the archwires. This is termed as “laceback side.” Laceback is not placed during the bond-up appointment, but 1 week after that (T_0_). Data collection for pain also commences after T_0_. This is to minimize the confounding effect of pain arising from initial archwire placement, which tends to subside in 1 week, as reported in the literature [31].

#### Standardization of Laceback Placement

Laceback is placed by the principal investigator using a preformed 0.010-inch stainless steel wire ligature (Ormco Corp). A trial on a study model fitted with Master Series, 0.022-inch slot, MBT prescription metal brackets (American Orthodontics) on the buccal segment was conducted to determine and standardize the method of securing a passive laceback.

**Figure 2.**
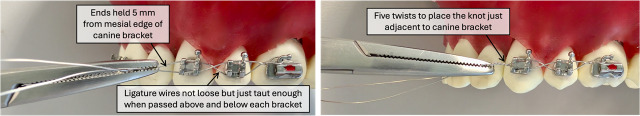
Trial on a study model to standardize the method of securing passive laceback.

First, the loop of the preformed long ligature was placed over the first molar hook. The ligature wire was then tied passively in a figure-of-eight pattern from the hook of the first permanent molar tube to the canine bracket of the same quadrant. At this stage, the ligature wires were not kept loose, but just taut enough as they passed above and below each bracket. Next, the free ends of the ligature wire were held together using Mathieu pliers at 5 mm from the mesial edge of the canine bracket. During the standardization process, it was determined that 5 twists of the ligature wire were required to position the knot just adjacent to the mesial wing of the canine bracket. This technique ensured that the laceback remained passive and was not overtightened, as shown in Figure 2. In addition, when lacebacks were placed on the participants, the gingiva surrounding the canine, premolar, and molar teeth was inspected to ensure that no blanching occurred, as this would indicate overtightening—a condition that this study aims to avoid. Finally, the excess wire was cut off using ligature cutters, leaving 3 mm of twisted wire (pigtail), which was tucked gingivally behind the canine bracket using a ligature tucker.

#### Interventions Common to Both Groups

The participants of this research undergo active orthodontic treatment at the postgraduate orthodontic clinic. The same bracket system, Master Series, 0.022-inch slot, MBT prescription metal brackets (American Orthodontics), and initial archwire (0.014-inch Euroform Super Elastic Nickel Titanium; Ortho-Care) are used for all participants. All brackets are bonded using the same standardized protocol, which involves 37% phosphoric acid etchant (Acid Etchant; American Orthodontics), light-cured adhesive primer (reliaBond Light-Cure Bonding Adhesive 5th Generation; Advanced Healthcare Ltd), and orthodontic composite resin (BracePaste Adhesive; American Orthodontics), following the manufacturer’s instructions. Extractions of all first or second premolars are done at least 2 weeks before the appointment for bond-up of orthodontic fixed appliances, so as to minimize pain arising from the extraction site that may confound the pain assessment. Archwires are ligated using elastomeric modules (EKSEN). The ends of the archwires are cut flush to the molar tubes and not cinched. Bite planes, intermaxillary elastics, headgear, or any auxiliaries are not used during the study period. The principal investigator delivers oral hygiene instructions, dietary instructions, and braces care instructions verbally with demonstration on a model using a standard script. An educational video on post-bond-up instructions is created and sent to the participants’ mobile devices for viewing. A patient information leaflet on brace care is also provided. All participants are supplied with a fluoridated toothpaste (Colgate Maximum Cavity Protection Fresh Cool Mint Toothpaste; Colgate-Palmolive Company) and a soft-bristle toothbrush (Colgate Zig Zag Toothbrush; Colgate-Palmolive Company). They are requested not to use any additional oral hygiene aids throughout the duration of the research. Participants undergo one round of scaling before lacebacks are placed during T_0_. Thereafter, they are informed not to go for professional teeth cleaning during the study period. Participants are reminded to check their braces for any dislodgement and breakage twice daily (morning and evening). If there are any orthodontic emergencies, participants are advised to call the clinic for an emergency appointment as soon as possible. Participants are also advised not to brush their teeth 2 hours before their appointments.

#### Control

**Figure 3.**
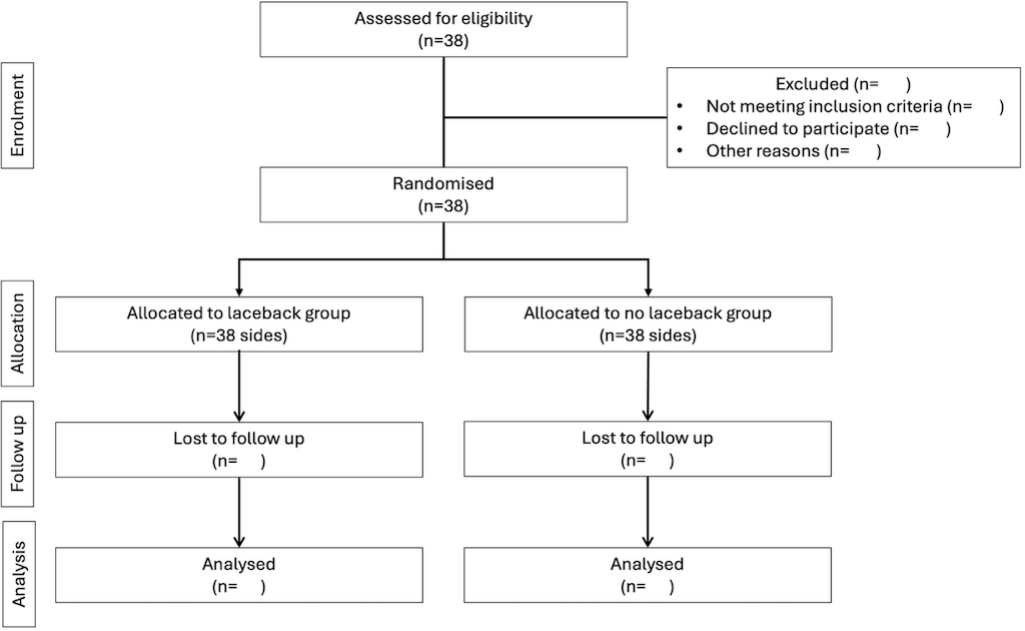
CONSORT (Consolidated Standards of Reporting Trials) flowchart.

On the maxillary and mandibular arches of the contralateral side, no laceback is placed. This serves as the control and henceforth is termed as “no laceback side.” Figure 3 explains the CONSORT flowchart for this study.

### Data Collection

#### Primary Outcome: Plaque Score

**Figure 4.**
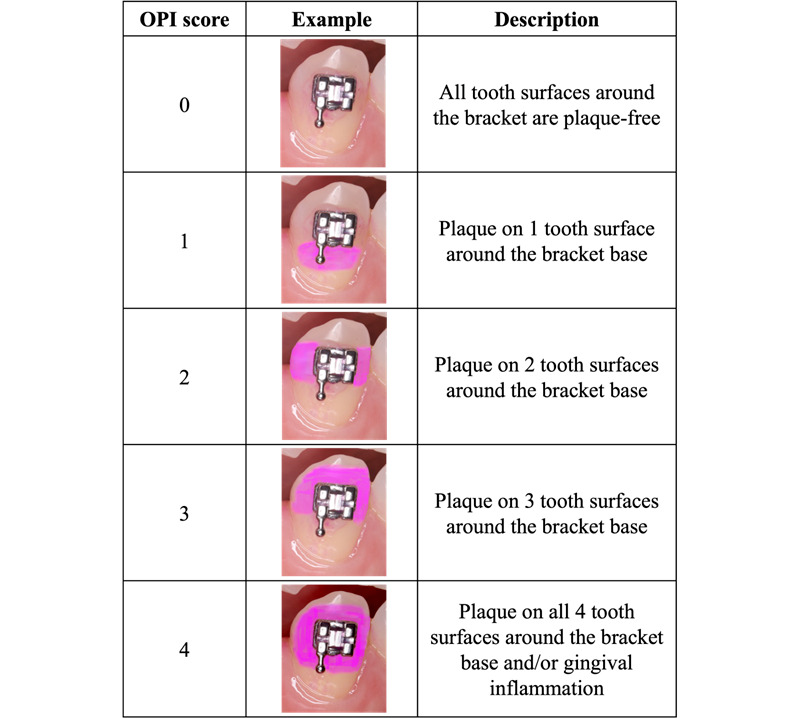
Criteria for Orthodontic Plaque Index scores.OPI: Orthodontic Plaque Index.

Plaque score is recorded to assess the patient’s oral hygiene status. For this study, the OPI by Beberhold et al [32] is chosen. The OPI is scored by visually examining whether dental plaque has accumulated at the tooth surface adjacent to each side of the bracket base (mesial, distal, occlusal or incisal, and gingival). The condition of the gingival margin is also noted. A score of 0 to 4 is given according to the criteria given in Figure 4. A higher plaque score indicates a poorer oral hygiene status.

Plaque score is recorded on the laceback and no laceback side during T_0_ (before laceback placement during first review appointment 1 wk after bond-up) as baseline, T_1_ (4 wks after laceback placement), and T_2_ (4 wks after T_1_). Once intervention starts at T_0_, if a participant cannot attend T_1_ or T_2_ appointments, a maximum of 2 weeks’ leeway is given, corresponding to the normal orthodontic follow-up interval of 6 weeks between appointments.

During T_0_, T_1_, and T_2_, archwires and lacebacks (if present) are removed, and plaque disclosing agent (GC Tri Plaque ID Gel; GC Asia Dental) is applied on the buccal surfaces of upper and lower canines, premolars, and first molars. Then, plaque score is recorded on those teeth by a blinded clinician not involved in the treatment of the participant. The tooth surface adjacent to each side of the bracket (mesial, distal, occlusal or incisal, and gingival) is visually examined for accumulation of plaque. The average OPI for the laceback and no laceback side is calculated by adding up the plaque score of all the teeth recorded on each side and dividing it by the number of teeth scored. The maximum plaque score for each side is 4, and the minimum score is 0. The criteria for scoring are explained in Figure 4 [32].

#### Examiner Calibration and Reliability Assessment

Inter- and intrarater reliability assessments of OPI were done before the trial commenced. Calibration of OPI was done on 20 orthodontic patients not participating in the trial. Using a Canon EOS RP digital camera (Canon Inc.) with RF100mm f/2.8L MACRO IS USM lens (Canon Inc.) and Godox Macro Ring Flash ML-150II (GODOX Photo Equipment Co., Ltd.) for illumination, buccal view intraoral photographs of these patients involved in the calibration exercise were also taken after application of plaque disclosing agent (GC Tri Plaque ID Gel; GC Asia Dental). Then, intrarater reliability was assessed by scoring 10 randomly selected photographs printed on photographic paper and rescoring them after 2 weeks. Reliability assessments were measured using Cohen kappa. The threshold was set to be more than 0.8 for this study, indicating good inter- and intrarater reliability [33].

#### Secondary Outcomes: Frequency of Archwire Complications

Archwire complications include the following:

1. Archwire fractured at any part distal to the canine bracket: counted as 1 incident.
2. Archwire dislodged from one or more canine brackets, premolar brackets, or first molar tubes: counted as one incident.
3. Archwire permanently deformed at any part distal to the canine bracket: counted as 1 incident.
4. Any combination of the above 3 situations is counted as 1 incident.
5. The whole archwire is lost: counted as 1 incident each on both laceback and no laceback sides.

The frequency of archwire complications on the laceback side and the no laceback side is recorded. The time points for recording are during T_1_, T_2_, and whenever a participant comes for an emergency appointment between T_0_ and T_2_. The number of complications on the upper and lower archwires is recorded separately and summed up for the laceback side and the no laceback side. During emergency appointments, dislodged archwires are religated using elastomeric modules and reinserted into the buccal tubes, whereas broken archwires are replaced by the same type of archwire. During T_1_ and T_2_ appointments, after data collection for archwire complications, archwires are removed to facilitate plaque score recording. After T_2_ data collection is completed, the treating clinician religates new or existing archwires according to their preference, as the trial would have ended.

#### Secondary Outcomes: Frequency of Laceback Complications

Laceback complications include the following:

1. Laceback ligature fractured at any part is counted as 1 incident.
2. Laceback ligature dislodged from 1 or more brackets of canine, premolar, and first molar hooks is counted as 1 incident.
3. A combination of the above 2 situations is counted as 1 incident.
4. The whole laceback is lost is counted as one incident.

Frequency of laceback complications is recorded. The time points for recording are during T_1_, T_2_, and whenever a participant comes for an emergency appointment between T_0_ and T_2_. Upper and lower lacebacks are visually examined during the appointment. The number of complications on the upper and lower lacebacks is recorded separately and summed up. The total number of laceback complications throughout the study period (T_0_ to T_2_) is recorded. During emergency appointments, dislodged lacebacks are retied from the first molar hook to the canine bracket using the same protocol described previously, whereas a broken or missing laceback is replaced using new but the same type of preformed 0.010-inch stainless steel wire ligature in the same manner. During T_1_ and T_2_ appointments, after data collection for laceback complications, lacebacks are removed to facilitate plaque score recording. After T_2_ data collection is completed, the treating clinician religates archwires with or without lacebacks according to their preference, as the trial would have ended.

#### Secondary Outcomes: Pain

Pain is recorded using the Visual Analog Scale, as it is the most widely used pain assessment scale and is suitable to be applied to the current age group of recruited participants. Pain score is recorded by the study participants on both laceback and no laceback sides. During T_0_, before placing lacebacks, the pain score is first recorded on both sides as baseline. Participants are given a pain diary to bring home during T_0_ and another copy during T_1_. At home, participants are reminded via mobile phone messaging (WhatsApp; Meta) to complete the pain diary. The pain diary given during T_0_ is collected back during T_1_ and T_2_. Participants are asked to rate the maximum pain experienced on each side during specific time points throughout the period between T_0_ and T_1_ and between T_1_ and T_2_. The time points are: 4 hours, 24 hours, 3 days, 1 week, 2 weeks, 3 weeks, 4 weeks from the review appointment. The 4-week time point can be recorded during the T_1_ or T_2_ appointment itself, or if the participant is unable to turn up for the appointments, they should record the pain score for the 4-week time point at home. Participants are instructed to place a mark along a 100-mm horizontal line at the most relevant position, whereby the left extreme indicates “no pain and discomfort” and the right extreme indicates “worst imaginable pain and discomfort.” Visual Analog Scale is measured to the nearest 1 mm using a standard metal ruler from the scale’s zero point to the patient’s mark and therefore quantified in a score with a range of 0 to 100. Each pain score reading is accompanied by an open-ended question asking what the cause of pain is (if any). Participants are informed that the pain recorded should arise from the laceback. Analgesics should not be taken prophylactically. If an analgesic is taken, the score should reflect the maximum pain experienced before taking the analgesic.

While there is a small risk of spill-over effect in this split-mouth study when recording pain score due to the occasional radiating nature of pain, this is minimized through patient education about pain localization. Prior to study commencement, participants will be explained about the different possible causes of pain during orthodontic treatment. Participants will be taught to look out for pain caused by the orthodontic appliance; for instance, long ligature wires rubbing on the cheek and twisted ends of laceback, which are pointed and poking on the oral mucosa. Participants are reminded to check their appliance in front of a mirror twice daily for breakages and identify areas on the left and right sides of their mouth that are sore from the orthodontic appliance.

### Blinding

The participant and the respective operators providing treatment could not be blinded due to the nature of the intervention. During T_1_, T_2_, or any emergency appointments, laceback complications are first assessed by the principal investigator. This is not blinded, as it is to merely record how many laceback complications occur on the side that has laceback, whereas the contralateral side does not have any laceback. The principal investigator then removes the laceback ligatures. Then, archwire complications and plaque scores are assessed by an orthodontic resident not treating the participant. As such, data collection for archwire complications and plaque scores will be blinded. As the lacebacks are removed by the principal investigator, the assessor is unaware of the site of intervention.

**Figure 5.**
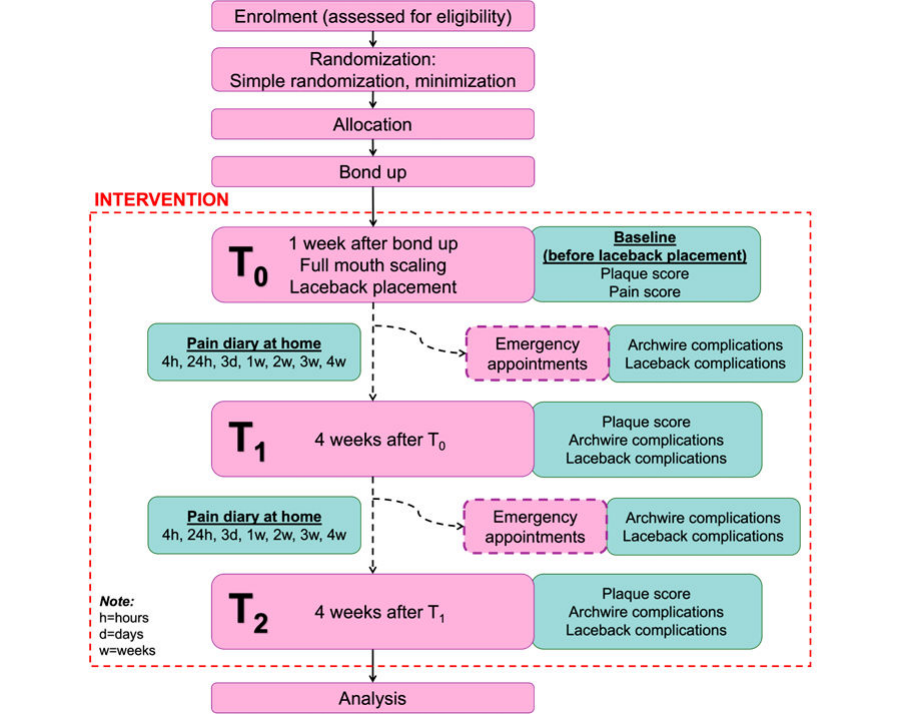
Overall study flow.

Examiner calibrations were conducted prior to trial commencement for plaque score assessment to ensure good inter- and intrarater reliability among all plaque score assessors. All data are coded and anonymized by the coinvestigator who withholds coding information about treatment allocation from the main data analyst until major analyses are completed. Unblinding is also allowed if a participant withdraws from the trial due to a severe adverse event. The overall study flow is illustrated in Figure 5.

### Intended Statistical Analysis

Statistical analysis is conducted using IBM SPSS Statistics (version 31). Descriptive statistics are used to report participant demographics, baseline characteristics, and frequency of laceback complications. To assess whether the data are normally distributed, interpreting the shape of the histogram and the Shapiro-Wilk test are used. Intra- and interrater reliability for plaque score assessment are measured using Cohen kappa. The threshold for good inter- and intrarater reliability is set to be more than 0.8.

Repeated outcome measurements of plaque scores will be analyzed using a mixed model for repeated measures. The model includes treatment group, time, and treatment-by-time interaction as fixed effects, with participant included as a random effect. A mixed model for repeated measures allows inclusion of participants with incomplete follow-up data without requiring formal imputation, assuming data are missing at random. If the data are not normally distributed, plaque scores over time (T_0_, T_1_, and T_2_) will be compared using the Friedman test.

From the pain diary gathered, the pain scores are plotted against time to allow calculation of area under the curve (AUC) using the trapezoidal rule [34]. Pain AUC will be calculated between T_0_ and T_1_ and T_1_ and T_2_. The mean pain AUC for laceback and no laceback sides will be calculated by averaging pain AUC values between T_0_ and T_1_ and between T_1_ and T_2_. To compare differences in mean pain AUC of laceback and no laceback groups, a paired *t* test is used if data are normally distributed. If the normality assumption is violated, the Wilcoxon signed-rank test is applied instead.

The mean frequency of archwire complications is recorded by summing up the number of complications that happen throughout the study period from T_0_ to T_2_. To compare differences in the mean frequency of archwire complications between the laceback and no laceback groups, a paired *t* test is used if data are normally distributed. The mean frequency of laceback complications and the number and percentage of participants with at least 2 laceback complications are also reported. The value of *P*<.05 is considered statistically significant.

Sensitivity analysis will be conducted by comparing the results of intention-to-treat and per-protocol analyses. If both analyses yield similar results, this demonstrates the robustness and validity of the study’s conclusions.

### Adverse Events

Some common complications during orthodontic fixed appliance treatment using laceback ligatures include pain, discomfort, and appliance breakage. These complications rarely require termination of orthodontic treatment and can be managed effectively using analgesics, orthodontic wax, and repairing the broken appliances when the situation arises. Nevertheless, an adverse events reporting form is prepared in case it is required by any of the study participants. During T_1_, T_2_, and emergency appointments, archwire and laceback complications are assessed, and any severe unintended harms beyond those listed in the protocol are recorded by the assessors. If a severe adverse event occurs, the laceback intervention is terminated for the participant. Further orthodontic management of the participant will be taken over by the treating clinician.

### Monitoring

A data monitoring committee is not deemed necessary for this study as it is of minimal risk. Trial monitoring is done on a 6-month basis through a research progress presentation conducted by the Research Management Committee of IIUM.

## Results

Ethical approval was granted by the IIUM Research Ethics Committee (IREC-2026‐582) in January 2026. Funding was secured through the IIUM Kulliyyah of Dentistry Postgraduate CHAIN Grant (CHAIN 26-014-0014) in the same month. Participant recruitment commenced in February 2026 with a planned completion date of June 2027. To establish measurement reliability, interrater and intrarater agreements were evaluated using Cohen kappa. Interrater reliability was calculated between 3 examiners (TLH, CCC, and SR) and a gold-standard examiner (SHN). The interrater Cohen kappa values ranged from 0.801 to 0.804. Intrarater reliability for each examiner demonstrated strong reproducibility, with Cohen kappa values ranging from 0.806 to 0.862.

The primary analysis is scheduled for completion by October 2027. Regardless of outcome, a paper reporting the primary results will be submitted for publication in a peer-reviewed orthodontic journal by June 2028. As of the submission of this protocol paper, 24 participants have been enrolled and 14 have completed all data collection visits. No deviations from the original protocol have been recorded.

## Discussion

This protocol describes a single-center, single-blind, split-mouth randomized controlled trial designed to evaluate the clinical and patient-reported outcomes of passive laceback ligatures during the alignment phase of orthodontic fixed appliance treatment. Existing evidence on laceback is almost entirely confined to its effects on incisor inclination and molar anchorage. A systematic review and meta-analysis found that laceback produced only a clinically negligible 0.50-mm incisor retroclination and a nonsignificant 0.45-mm greater molar mesialization compared to controls [5]. In that review, the authors explicitly flagged the absence of data on oral hygiene, appliance breakages, and pain as critical gaps warranting future investigation. This trial directly addresses all three of those gaps, making it the first RCT to do so within a single, methodologically controlled study. In contrast to prior studies, which used parallel-group designs and cephalometric outcome measures, this protocol uses a split-mouth design and focuses exclusively on outcomes that have been identified as both clinically meaningful and patient-relevant.

Based on the null hypothesis that laceback will not produce statistically significant differences in clinical and patient-reported outcomes, we anticipate that this trial may reveal that passive laceback ligatures are associated with modestly elevated plaque scores compared to the no-laceback side, reflecting the additional plaque-retentive surface created by the figure-of-eight ligation pattern. We hypothesize that the laceback side may also demonstrate a lower frequency of archwire complications, lending quantitative support to the widely held but as yet unverified clinical belief that lacebacks help stabilize flexible archwires against masticatory forces. With respect to pain, we hypothesize that the laceback side may be associated with a small but measurable increment in discomfort, particularly in the early weeks following placement, which may have implications for patient counseling.

When laceback was first introduced, it was intended to counteract the mesial crown tip of the canine bracket and prevent labial segment proclination during the leveling and alignment phase of preadjusted edgewise therapy. Although the effectiveness of laceback in controlling the anteroposterior position of the labial segment has since been disputed, it continues to be used by clinicians for several other purposes, most notably the protection of flexible initial archwires from masticatory forces [6]. The role of laceback in supporting the main archwire, however, remains largely anecdotal. If laceback is demonstrated to reduce the frequency of archwire breakage and dislodgement, it may serve as a valuable tool in minimizing treatment disruptions and unplanned emergency appointments, both of which impose additional costs and inconveniences on patients and clinicians alike. Conversely, if it does not meaningfully contribute to archwire stability, the justification for its routine use would need to be reconsidered.

Orthodontic fixed appliances and their auxiliaries are recognized as plaque-retentive environments, providing additional surface area for biofilm formation and impeding effective oral hygiene. Maintaining adequate oral hygiene is of paramount importance during fixed appliance therapy to minimize iatrogenic complications such as enamel demineralization, dental caries, and periodontal deterioration [35,36]. By evaluating the effects of laceback on a patient’s oral hygiene, this research will provide valuable insights to help clinicians decide whether additional oral hygiene instructions or preventive measures are necessary when using laceback. This will benefit patients by ensuring good oral health throughout the treatment process and contributing to improved overall treatment outcomes.

Furthermore, pain and discomfort are common concerns during orthodontic fixed appliance treatment. As an additional appliance component in direct contact with the canine bracket and molar hook, laceback may contribute to soft tissue irritation, altered force distribution, and heightened sensitivity, particularly during the first days after placement. However, the specific contribution of laceback to pain during the alignment phase has not previously been investigated, leaving clinicians without evidence to inform patient counseling at the time of consent.

This study also reflects the broader shift in orthodontic research toward patient-reported outcome measures as primary or coprimary endpoints. Recent studies have emphasized that orthodontic evidence should prioritize outcomes that are directly meaningful to patients, not merely clinician-assessed clinical indices [37-39]. By incorporating pain and discomfort as primary endpoints alongside plaque score and appliance complication rates, this trial provides the more comprehensive evidence base required for evidence-based clinical decision-making and patient-centered consent.

Key strengths of this protocol include its randomized split-mouth design, which controls for patient-level confounders; the use of validated and orthodontically appropriate outcome measures (OPI for plaque and Visual Analog Scale for pain); standardized laceback placement verified against a study model prior to commencement; blinded plaque score assessment; and prospective trial registration (ClinicalTrials.gov ID: NCT07039071). The split-mouth design is particularly advantageous in this context because the intervention is strictly localized: the presence of a laceback on 1 side of the mouth does not mechanically or biologically influence plaque accumulation, archwire stability, or pain on the contralateral side, thereby satisfying the key prerequisite for a valid split-mouth trial with negligible carry-across effect.

Several limitations should be acknowledged. First, the single-center nature of this study, conducted within a specialist postgraduate orthodontic clinic, may limit generalizability to primary care settings or to practices using different bracket systems or prescriptions. Second, the inability to blind participants or the treating clinician to the intervention side is an inherent limitation of the split-mouth design; this risk has been partially mitigated by implementing blinded plaque score assessment. Third, the 8-week follow-up period may not capture longer-term changes in oral hygiene adaptation or pain habituation. Fourth, the use of a single operator for laceback placement enhances consistency and reproducibility within this trial, but may restrict external validity across different clinical hands. These limitations will be transparently reported in the subsequent efficacy paper and will be considered carefully during interpretation of results.

If significant effects of laceback on oral hygiene or pain are demonstrated, future work should explore whether targeted patient education delivered at the time of laceback placement can mitigate these adverse effects. Multicenter replication across different clinical settings, bracket systems, and operator experience levels would strengthen external validity and test the generalizability of these findings. Future trials might also extend the follow-up period beyond 8 weeks to encompass the full alignment and leveling phase and to determine whether adverse effects on oral hygiene or pain attenuate over time as patients adapt. Additionally, investigation of modified laceback designs, such as the fully twisted or modified anchorage configurations described in the literature, could determine whether specific laceback types differ in their complication profiles.

The findings from this trial will be disseminated through a peer-reviewed publication in an orthodontic or dental journal irrespective of outcome, in keeping with the principles of complete and transparent clinical trial reporting. Results will also be presented at national and international orthodontic conferences. The dissemination strategy will prioritize journals offering open-access publishing options to maximize accessibility, particularly for clinicians and researchers in low-resource settings where evidence-based guidance on auxiliary use is most critically needed. A lay summary of findings will be made available to all participants upon request, consistent with the patient information and consent framework provided at enrollment.

## Supplementary material

10.2196/88793 Checklist 1 Standard Protocol Items: Recommendations for Interventional Trials (SPIRIT) 2025 checklist.

## Abbreviations

AUC: area under the curve
CONSORT: Consolidated Standards of Reporting Trials
IIUM: International Islamic University Malaysia
MBT: McLaughlin, Bennett, and Trevisi
MMRM: mixed model for repeated measures
OPI: Orthodontic Plaque Index
PRO: patient-reported outcome
SPIRIT: Standard Protocol Items: Recommendations for Interventional Trials
